# Supplementary figures and images for: Empirical aesthetics of bridges
Source: PLoS One. 2025 Dec 18;20(12):e0338493. doi: 10.1371/journal.pone.0338493 (PMC12714226; doi:10.1371/journal.pone.0338493)

**S1 Fig. Scree Plot for Experiment 1.**

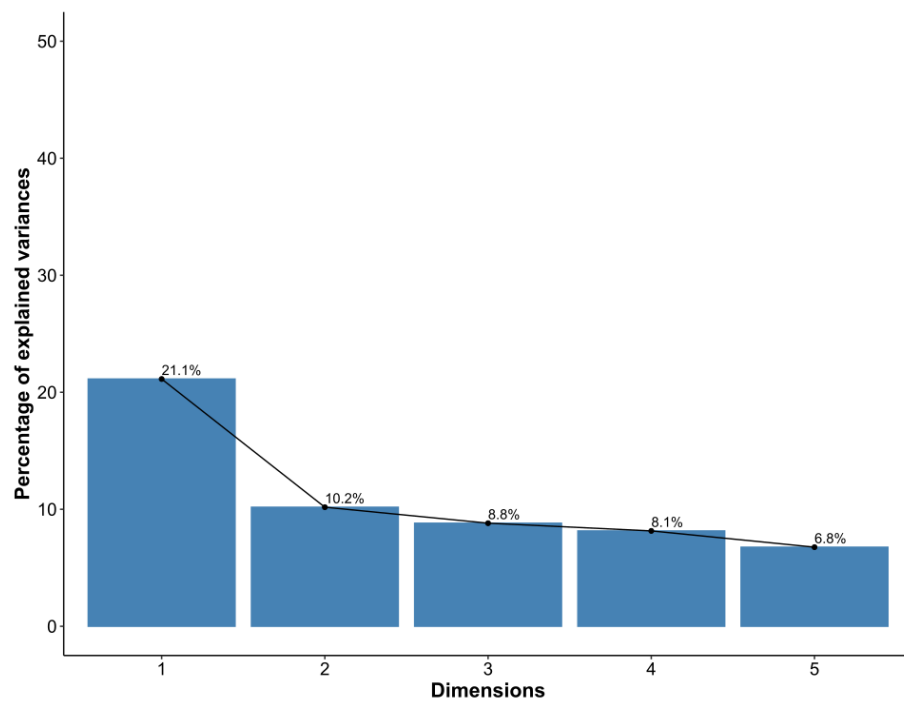

Supplement: S1 Fig — (PDF) [file pone.0338493.s001.pdf]

**S2 Fig. Scree Plot for Experiment 2.**

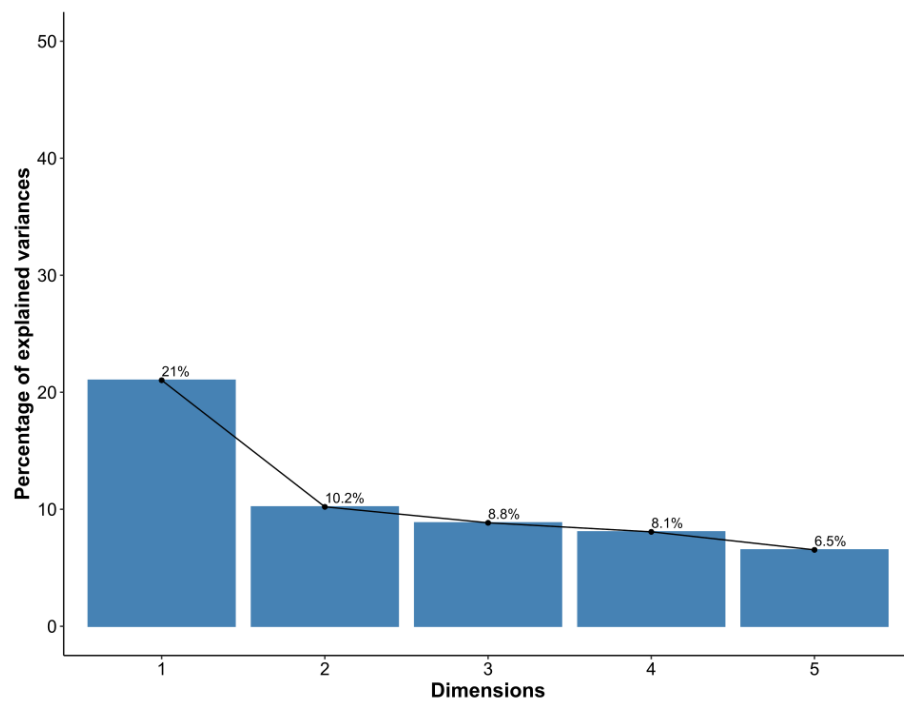

Supplement: S2 Fig — (PDF) [file pone.0338493.s002.pdf]

**S3 Fig. Correlation Matrix of Aesthetic Ratings for Experiment 1.**

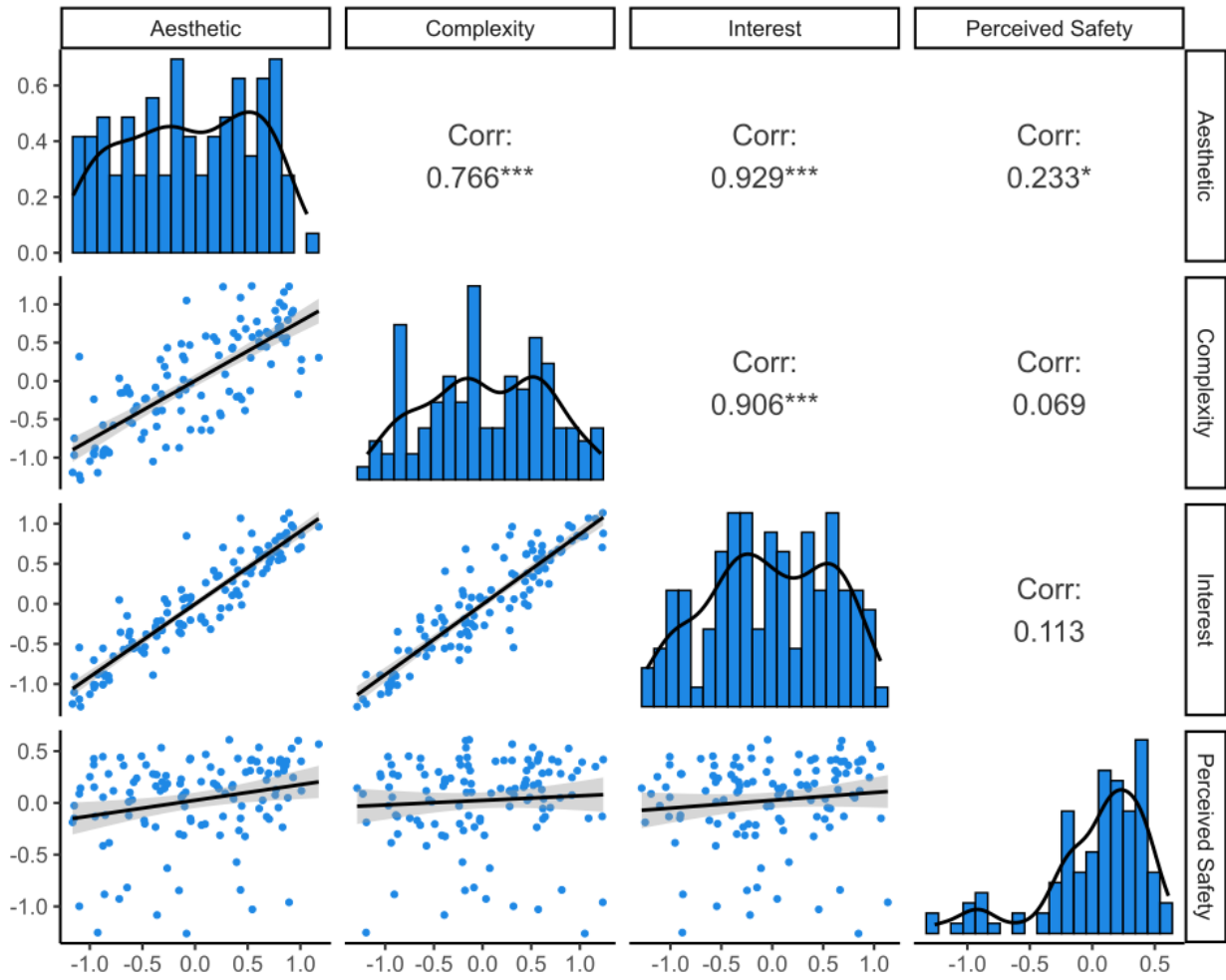

Supplement: S3 Fig — (PDF) [file pone.0338493.s003.pdf]

**S4 Fig. Correlation Matrix of Aesthetic Ratings of Experiment 2.**

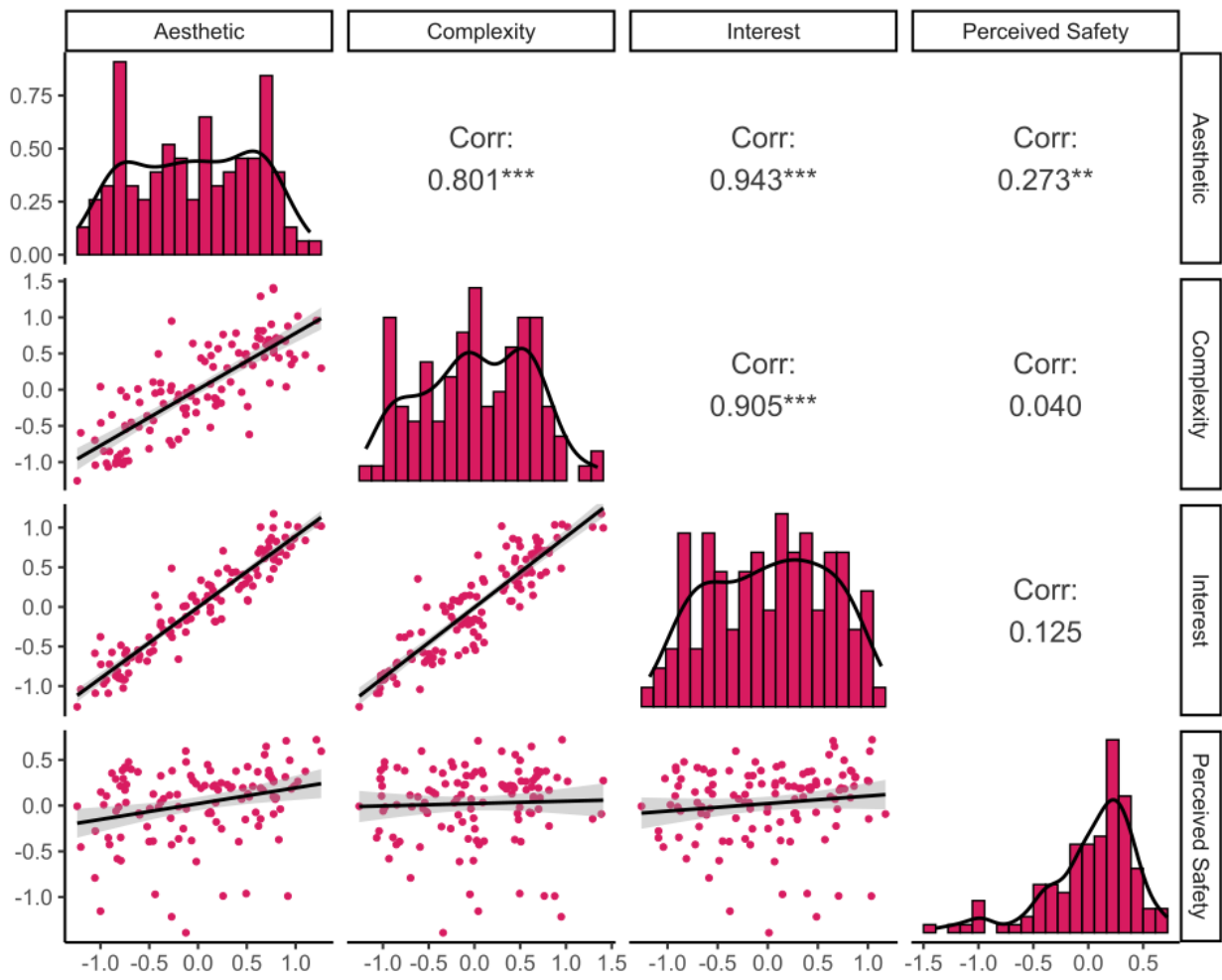

Supplement: S4 Fig — (PDF) [file pone.0338493.s004.pdf]

**S5 Fig. Familiarity Distribution of Bridges in Experiment 1 and 2.**

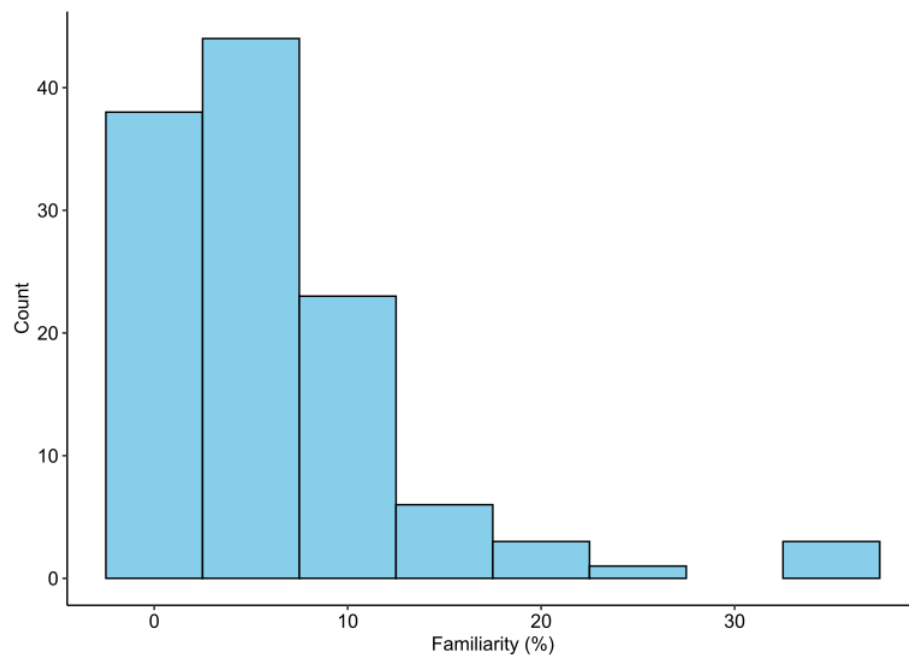

Supplement: S5 Fig — (PDF) [file pone.0338493.s005.pdf]
