# Supplementary material for: Empirical aesthetics of bridges
Source: PLoS One. 2025 Dec 18;20(12):e0338493. doi: 10.1371/journal.pone.0338493 (PMC12714226; doi:10.1371/journal.pone.0338493)
Supplement: S1 Table — (PDF) [file pone.0338493.s006.pdf]

S1 Table. Contribution of Variables to Dimension 1 and 2 of Experiment 1.

Variable	Dimension 1	Dimension 2
Aesthetic	20.96%	0.37%
Complexity	18.27%	3.48%
Interest	22.04%	0.14%
Perceived Safety	0.98%	5.32%
Type	17.40%	17.20%
Bridge Depth	8.75%	1.72%
Material	5.69%	35.91%
Aesthetic Premium	4.89%	12.27%
Age	1.02%	23.57%
