# Supplementary material for: Empirical aesthetics of bridges
Source: PLoS One. 2025 Dec 18;20(12):e0338493. doi: 10.1371/journal.pone.0338493 (PMC12714226; doi:10.1371/journal.pone.0338493)
Supplement: S2 Table — (PDF) [file pone.0338493.s007.pdf]

S2 Table. Contribution of Variables to Dimension 1 and 2 of Experiment 2.

Variable	Dimension 1	Dimension 2
Aesthetic	21.46%	0.28%
Complexity	18.74%	3.20%
Interest	21.89%	0.19%
Perceived Safety	1.08%	6.40%
Type	17.14%	17.85%
Bridge Depth	8.82%	2.39%
Material	5.48%	35.89%
Aesthetic Premium	4.54%	11.30%
Age	0.86%	22.49%
