# Supplementary material for: Empirical aesthetics of bridges
Source: PLoS One. 2025 Dec 18;20(12):e0338493. doi: 10.1371/journal.pone.0338493 (PMC12714226; doi:10.1371/journal.pone.0338493)
Supplement: S1 Text — (PDF) [file pone.0338493.s008.pdf]

Participant Demographics and Professional Background for Experiment 1.

N	Age (Mean ± SD)	Female	Male	Architecture Experience	Engineering Experience
98	25.23 ± 3.55	40 (40.8%)	55 (56.1%)	13 (13.3%)	35 (35.7%)

Country of Residence Distribution for Experiment 1 Participants.

Country of Residence	N	%
Portugal	26	26.5%
South Africa	17	17.3%
Mexico	13	13.3%
Poland	12	12.2%
United Kingdom	7	7.1%
Canada	5	5.1%
Greece	3	3.1%
Germany	2	2.0%
Israel	2	2.0%
Italy	2	2.0%
Chile	1	1.0%
France	1	1.0%
Hungary	1	1.0%
Ireland	1	1.0%

Country of Residence	N	%
Latvia	1	1.0%
Netherlands	1	1.0%
Slovenia	1	1.0%
Spain	1	1.0%
Not reported	1	1.0%

Participant Demographics and Professional Background for Experiment 2.

N	Age (Mean ± SD)	female	male	Architecture Experience	Engineering Experience
92	25.18 ± 3.74	47 (51.1%)	43 (46.7%)	5 (5.4%)	30 (32.6%)

Country of Residence Distribution for Experiment 2 Participants.

Country of Residence	N	%
Portugal	19	20.7%
Poland	18	19.6%
Mexico	15	16.3%
South Africa	13	14.1%
Chile	5	5.4%
Greece	5	5.4%
Hungary	3	3.3%

Country of Residence	N	%
United Kingdom	3	3.3%
Spain	2	2.2%
Czech Republic	1	1.1%
Estonia	1	1.1%
France	1	1.1%
Ireland	1	1.1%
Japan	1	1.1%
Netherlands	1	1.1%
Sweden	1	1.1%
United States	1	1.1%
Not reported	1	1.1%

Participant Demographics and Professional Background for Experiment 3.

N	Age (Mean ± SD)	Female	Male	Architecture Experience	Engineering Experience
49	25.00 ± 4.33	20 (40.8%)	27 (55.1%)	3 (6.1%)	13 (26.5%)

Note on Experiment 3: Due to a technical error, some participants' IDs were not properly recorded. Since we did not collect data on country of residence within the experiment itself, we cannot provide the breakdown for Experiment 3 as it requires subject-ID matching with Prolific's records.
