# Supplementary material for: Empirical aesthetics of bridges
Source: PLoS One. 2025 Dec 18;20(12):e0338493. doi: 10.1371/journal.pone.0338493 (PMC12714226; doi:10.1371/journal.pone.0338493)
Supplement: Attachment — Submitted filename: Response to Reviewers.docx [file pone.0338493.s010.docx]

Dear Dr. Matteo Bodini,

Thank you for the opportunity to submit our revised manuscript. We address reviewers’ comments below in blue. Any additions to the manuscript are also included below in orange.

On behalf of all authors,

Claudia Damiano

**Reviewer #1:** Summary
This manuscript presents a comprehensive, well-designed, and highly original empirical investigation of the aesthetic perception of bridges. Drawing from a dataset of 318 real-world bridges, rated by over 250 participants across multiple dimensions (aesthetic liking, complexity, interest, perceived safety), the authors identify two latent perceptual dimensions—Aesthetic and Perceived Safety—and examine how they relate to both structural design features and mid-level visual properties. The study integrates civil engineering expertise with cognitive science methods and contributes a publicly available dataset for future research.
The topic is timely and underexplored, and the study is methodologically sound and theoretically significant. The writing is clear, the analyses are appropriate and replicable, and the discussion is thoughtful and well contextualized.

Dear Dr. Ivan Stojilovic,

Thank you for the kind words and thank you for taking the time to review our manuscript. We address your comments and questions below.

Major Comments
• The manuscript explains that design features were annotated by experts, but it is not clear whether one or multiple annotators performed the coding. If multiple coders were involved, inter-rater reliability (e.g., Cohen’s κ) should be reported. If a single expert conducted the annotations, this should be stated explicitly, along with a brief justification (e.g., relevant experience or qualifications).

Apologies for the lack of detail regarding the annotation procedure. We have now added the following to the Methods section (lines 196-201):

These annotations were made by an engineering student working under very close supervision from an expert (author P.G.), an experienced bridge engineer, based on extensive knowledge of past and current design practice. The student and expert first annotated several images together, and the expert answered any questions from the student regarding the task. The student then annotated the entire set of images. Following this, author P.G. then reviewed all annotations.

• While the authors rightly emphasize that their study captures laypeople’s perspectives, it would be helpful to acknowledge that aesthetic preferences may vary across cultures. The discussion could be strengthened by briefly addressing the potential role of cultural context—e.g., whether individuals from Asia, Europe, or Africa might evaluate bridge aesthetics differently—and what that might imply for the generalisability of the findings.

This is a great point, thank you. In addressing another reviewer’s comment, we note that the participants in our study were born and/or are residing in countries across the world, not just in North America. In fact, the majority of participants reside in countries outside of North America, suggesting that these aesthetic judgements may be generalizable across cultural contexts. However, because we do not have sufficient data to actually perform comparisons across countries in the current study, we have added the following to the Discussion section (lines 662-681), addressing the potential role of culture in evaluation of bridge aesthetics:

Additionally, while our image set includes bridges from around the world, and our participants reside in countries around the world as well, we lack sufficient data for comparisons of aesthetic evaluations across cultures. Cultural background has been shown to shape aesthetic responses to artworks [50] and everyday objects [51], thus follow-up studies should explore whether individuals from different cultural regions evaluate bridge aesthetics differently. Understanding how cultural context interacts with visual and structural design features will be crucial for improving the generalizability and cross-cultural applicability of our findings.

• The finding that angularity positively predicts aesthetic responses contradicts a large body of literature in empirical aesthetics. While the authors offer plausible and insightful explanations (e.g., domain-specific aesthetics of structural engineering, inferred size and complexity), these remain speculative. The discussion could be enhanced by suggesting concrete follow-up experiments to disentangle these interpretations.

Thank you for this suggestion. This finding should indeed be further explored in follow-up studies. We have now added concrete follow-up plans to our Discussion (see lines 621-624):

More work should be carried out to better understand how such factors may influence the aesthetics of bridges. For instance, follow-up studies to determine whether observers have the ability to infer span length from the 2D image, and whether that is related to their sense of awe, aesthetic pleasure, and perceived creativity of the design, could distinguish between these possible explanations, or find that they are both valid to some extent.

Minor Comments:
• Missing p-value (line 518): In reporting the results of Experiment 3, the p-value for interest is missing: “interest: t(49) = -33.52;”
Please add the corresponding p-value for consistency (presumably p < 0.001).
• Typographical error (line 618): The word “For” is incorrectly capitalised in the middle of the sentence
• While the diversity of bridges is mentioned, a brief breakdown of bridge distribution by type, material, age, aesthetic premium and region (if available) would help readers assess the representativeness of the dataset.

Thank you for pointing out these omissions/typos. These have now been fixed. Regarding the last point, this information is available within the .csv files available on OSF (e.g., exp1_data.csv), including each bridge’s official name, which can be used to find the region. We have now expanded a section of the Methods which states where this breakdown of the design features can be found (see lines 206-213):

Along with the images, we provide a .csv file (e.g., exp1_data.csv) with the ratings of the four scales for Experiment 1 (liking, visual complexity, interest, perceived safety) and five scales for Experiment 2 (liking, visual complexity, interest, perceived safety, familiarity). The rating files also include the design features of the bridges. These design features include the five described above, along with the official name of the bridge, which can also be used to determine the bridge’s location. The BDA (Bridge Design and Aesthetics) data set is freely available to researchers via the Open Science Framework (<https://osf.io/wf5qg/?view_only=937d90e0271348f6ae313a1cac2a1062>).

Conclusion
This is an important and methodologically rigorous contribution to the growing field of empirical aesthetics. It advances our understanding of how large-scale engineered structures are perceived, opens new directions for interdisciplinary research, and provides a high-quality dataset for future studies. With minor revisions as suggested above, I strongly recommend this manuscript for publication.

Thank you again for the positive review and helpful comments.

**Reviewer #2:** REJECTED. This is trivial disorganized and immature work both technically/analytically and structurally. Research work without ‘conclusion’???

The lack of innovation of this work in the science border is concrete!! The main novelty?? characterized contribution??? It simply can be seen from superficial review and all outdated references, with at least 3 years gap!!!!

There are many untreated technical concerns: The framework is a simple straight image analysis without any clear modeling procedure/proper justifications/lacking substantive enhancements to bolster the theoretical underpinnings and methodological robustness of the study/feature extraction strategy, noise removal/deep discussion about the claimed ‘dimension’, ‘safety’…

The prerequisite of this draft is the use of a unified pixel size and therefore, any modification to input data such as changes in resolution (pixels, grid structure, …) compromises data integrity. Hence, the capability and stability approval of the given model is not certified.

Incohesive English/inconsistent with the third passive/frequent flaws…

Unconvincing and documented Discussion/technical limitations/solid comparison with other scholars’ approaches/constraints in getting features/…

If and only if we consider Fig 10, it is simple uncertainty quantification where in comparison with advanced approaches like <https://link.springer.com/article/10.1007/s00366-023-01852-5>... It is uncompetitive

Ill-formatted references in terms of required identifiers

We are very sorry to hear that the work did not meet your expectations, but we appreciate the opportunity to clarify our goals and methodology.

This project was a collaboration between researchers in cognitive psychology/empirical aesthetics and bridge engineering. Our contribution is that we are providing the first systematic dataset and analysis of how laypeople evaluate the aesthetics of real-world bridges, which is an understudied but important topic across both fields.

Our approach used validated techniques from empirical aesthetics, such as participant ratings, visual feature extraction, and multivariate analysis, to explore how design features relate to perceptions of beauty, interest, complexity, and safety. We understand that this is not a traditional engineering modeling paper, but it is not intended to be. Instead, it serves as a foundation for future interdisciplinary work on how public infrastructure may impact people’s aesthetic experiences within their daily environments.

We hope this clarifies our intent and the value of our contribution within this context.

**Reviewer #3:** Clarity of Methods: The paper is well structured and clearly written. One area that could use a bit more detail is the description of the Factorial Analysis of Mixed Data (FAMD). A brief explanation of why FAMD was chosen over other dimensionality reduction methods would help readers less familiar with the technique.

Thank you for the kind words about our paper. Regarding the description and justification of using FAMD, we already provide this on lines 283-301. FAMD is used when there are both continuous and categorical variables in the dataset.

The presence of multiple continuous and categorical variables in our data necessitates the use of a multivariate statistical analysis. To that end, we employed Factorial Analysis of Mixed Data (FAMD) to understand the relationship between the subjective ratings of bridges and their design features [25,26]. FAMD is a type of dimensionality reduction technique suitable for datasets containing both continuous and categorical variables. It can be considered as a combination of principal component analysis (PCA) and multiple correspondence analysis (MCA). Specifically, FAMD starts by converting categorical variables into indicator matrices and combines them with the standardized continuous variables. Then, FAMD performs PCA on the transformed data to determine principal components. PCA identifies the directions (principal components) along which the variance in the data is maximized, thereby reducing redundancy and highlighting the most important features of the data. This is particularly important when quantitative variables are highly correlated (see **Figures S3 and S4)**. In addition to the usual benefits of dimensionality reduction, FAMD is particularly useful in our analysis for several reasons. First, it enables us to visualize and compare the contribution of each variable to the overall variance within the data. Second, FAMD facilitates meaningful comparisons both among and within categorical and continuous variable sets while preserving their original data types. Third, by representing each observation on the factor map, FAMD allows for comparisons among individual bridges. All data analysis was conducted using the R statistical software (R version 4.1.3). FAMD was performed using the FAMD function from the FactoMineR package [27].

Participant Characteristics: The study includes ratings from 254 participants, which is a strong sample size. It would strengthen the paper to provide a more detailed demographic breakdown (e.g., cultural or professional background), since perceptions of aesthetics and safety can vary across populations.

Thank you for this good suggestion. We have now included breakdowns of participants’ architecture/engineering experience and country of residence in the supplementary materials (see Tables S3-S7) and have expanded our Discussion section (see lines 662-681), addressing the potential role of culture in evaluation of bridge aesthetics:

Additionally, while our image set includes bridges from around the world, and our participants reside in countries around the world as well, we lack sufficient data for comparisons of aesthetic evaluations across cultures. Cultural background has been shown to shape aesthetic responses to artworks [50] and everyday objects [51], thus follow-up studies should explore whether individuals from different cultural regions evaluate bridge aesthetics differently. Understanding how cultural context interacts with visual and structural design features will be crucial for improving the generalizability and cross-cultural applicability of our findings.

Practical Implications: The findings on bridge type and material are compelling. Expanding the discussion slightly on how these insights might inform future design practices or policy guidelines could enhance the paper’s practical relevance.

Thank you for this suggestion. We have now added a paragraph to the Discussion to address the practical relevance of our findings (lines 634-641).

Our insights about which bridge types are preferred could be useful for informing design decisions in contexts where public engagement or visual impact are important considerations. For example, city planners might consider incorporating the preferred bridge types into highly visible or frequented areas within a city. Conversely, when visual impact is a secondary consideration, more economical or simpler bridge types may be more appropriate. Additionally, our findings on material and perceived safety may guide policy considerations around heritage preservation, since older stone bridges were perceived as safer and more appealing, potentially justifying their maintenance in urban planning contexts.

Perceived vs. Actual Safety : Since perceived safety emerged as a key dimension, it may be helpful to briefly connect this with research on actual safety outcomes. For example, 10.1080/23249935.2025.2516817. While focused on road safety, it shows how infrastructure features translate into measurable safety risks. Referencing such work could broaden the discussion by linking perceptions with outcome-based evidence.

This is a good point, and thank you for the reference. We now reference this work and discuss how our perceived safety finding could be linked to actual safety outcomes in the future (lines 557-564).

While our study focused on subjective perceptions of safety, work in transportation research has linked features of the environment with measurable safety outcomes [34]. For example, roadway factors (e.g., speed limit), lighting (e.g., daylight), and structural elements (e.g., urban vs. rural) have been shown to influence crash risk and pedestrian injury. Future research could explore whether bridge features that are intuitively associated with safety are similarly linked to objective safety outcomes or incident rates. This connection between perceived and actual safety could inform both public communication and design practices for new infrastructure.
